# Entropy and Time

**DOI:** 10.3390/e22040430

**Published:** 2020-04-10

**Authors:** Arieh Ben-Naim

**Affiliations:** Department of Physical Chemistry, The Hebrew University of Jerusalem, Jerusalem 91904, Israel; ariehbennaim@gmail.com

**Keywords:** arrow of time, entropy, Shannon’s measure of information, second law of thermodynamics, H-theorem, H-function

## Abstract

The idea that entropy is associated with the “arrow of time” has its roots in Clausius’s statement on the Second Law: “*Entropy of the Universe always increases*.” However, the explicit association of the entropy with time’s arrow arises from Eddington. In this article, we start with a brief review of the idea that the “increase in entropy” is somehow associated with the direction in which time increases. Then, we examine three different, but equivalent definitions of entropy. We find that none of these definitions indicate any hint of a relationship between entropy and time. We can, therefore, conclude that entropy is a timeless quantity. We also discuss the reasons as to why some scientists went astray in associating entropy with time’s arrow. Finally, we shall discuss Boltzmann’s H-Theorem, which is viewed by many as a proof of the Second Law of Thermodynamics.

## 1. Introduction: Three Different but Equivalent Definitions of Entropy

Open any book which deals with a “theory of time,” “time’s beginning,” and “time’s ending,” and you are likely to find the association of entropy and the Second Law of Thermodynamics with time [1,2,3,4,5,6,7,8,9,10,11,12,13,14,15,16,17]. The origin of this association of “Time’s Arrow” with entropy can be traced to Clausius’ famous statement of the Second Law [18]: “*The entropy of the universe always increases”.* The explicit association of entropy with time is due to Eddington [19], which we shall discuss in the next section.

The statement “*entropy always increases,*” implicitly means that “*entropy always increases with time*”. In this article, we show that statements like “*entropy always increases*” are meaningless; entropy, in itself does not have a numerical value. Therefore, one cannot claim that it increases or decreases. Entropy changes are meaningful only for well-defined thermodynamic processes in systems for which the entropy is defined.

In this Section we briefly present three different, but equivalent definitions of entropy. By “different” we mean that the definitions do not follow from each other, specifically, neither Boltzmann’s, nor the one based on Shannon’s measure of information (SMI), can be “derived” from Clausius’s definition.

By “equivalent” we mean that, for any process for which we can calculate the change in entropy, we obtain the same results by using the three definitions. Note carefully, that there are many processes for which we cannot calculate the pertinent, entropy changes. Therefore, we cannot claim equivalency in general. However, it is believed that these three definitions are indeed equivalent although no formal proof of this is available.

At this point, it is appropriate to note that there are many other “definitions” of entropy, which are not equivalent to either one of the three definitions discussed in this article. I will not discuss these here, since they were discussed at great length in a several previous publications [20,21,22,23,24,25,26,27,28,29].

In the following sections we shall introduce three definitions of entropy. The first definition originated in the 19th century, stemming from the interest in heat engines. The introduction of entropy into the vocabulary of physics is attributed to Clausius. In reality, Clausius did not define entropy, but rather only changes in entropy. Clausius’s definition, together with the Third Law of Thermodynamics, led to the calculation of “absolute values” of the entropy of many substances.

The second definition is attributed to Boltzmann. This definition is sometimes referred to as either the microscopic definition of entropy. It relates the entropy of a system to the total number of accessible micro-states of a thermodynamic system characterized macroscopically by the total energy *E*, volume *V*, and total number of particles *N* [for a multi-component system *N* may be reinterpreted as the vector $\left(N_{1},\ldots ,N_{c}\right)$, where $N_{i}$ is the number of atoms of type *i*]. The extension of Boltzmann’s definition to systems characterized by independent variables other than the (E,V,N) is attributed to Gibbs.

The Boltzmann definition seems to be completely unrelated to the Clausius definition. However, it is found that, for all processes for which entropy changes can be calculated by using Boltzmann’s definition, the results agree with entropy changes calculated using Clausius’s definition. Although, there is no formal proof that Boltzmann’s entropy is equal to the thermodynamic entropy, as defined by Clausius, it is widely believed that this is true.

The third definition, which I will refer to as the ABN’s definition, is based on Shannon’s measure of information (SMI) [22,24]. It may also be referred to as the microscopic definition of entropy. However, this definition of the entropy is very different from Boltzmann’s definition. This is also the only definition which provides a simple, intuitive, and meaningful interpretation of entropy and the Second Law.

We also note, that calculations of entropy changes, based on the SMI definition, agree with those calculations based on Clausius’s, as well as on Boltzmann’s definition. Unlike Boltzmann’s definition however, the SMI definition does not rely on calculations of the number of accessible states of the system. It also provides directly the entropy function of an ideal gas, and by extension, also the entropy function for a system of interacting particles.

In each of the following sections, we shall examine the question of the “range of applicability” and of the possible time-dependence of entropy. As we shall see, the independence of entropy on time is most clearly revealed from the SMI-based definition of entropy. At this point it is appropriate to quote Einstein on Thermodynamics:

“It is the only physical theory of universal content, which I am convinced, that within the framework of applicability of its basic concepts will never be overthrown.”

I fully agree that thermodynamics will never be overthrown. This quotation features in numerous popular science books. It is ironic that most of the authors who quote Einstein on thermodynamics emphasize the word “overthrown,” but ignore Einstein’s words “framework of applicability.” These are exactly the words that many authors overlook when writing about the entropy and the Second Law. They apply the concept of entropy and the Second Law in realms where they do not apply.

In this article, I will discuss the misconstrued association of Entropy with time. As we shall see, the cause for this association of entropy with time is that thermodynamics was used not “*within the framework of its applicability*”.

### 1.1. Clausius’s “Definition” of Entropy

I enclose the word “definition” in inverted commas because Clausius did not really define entropy. Instead, he defined a small change in entropy for one very specific process. It should be added that, even before a proper definition of entropy was posited, entropy was confirmed as a state function*,* which means that whenever the macroscopic state of a system is specified, its entropy is determined [20,21,22,23,24,25,26,27,28,29].

Clausius’s definition, together with the Third Law of Thermodynamics led to the calculation of “absolute values” of the entropy of many substances.

Clausius started from one particular process; the spontaneous flow of heat from a hot to a cold body. Based on this specific process, Clausius defined a new quantity which he called Entropy. Let dQ>0 be a small quantity of heat flowing *into* a system, being at a given temperature *T*. The change in entropy is defined as:

(1) $$dS=\frac{dQ}{T}$$

The letter *d* stands for a very small quantity. *T* is the absolute temperature, *Q* has the units of energy, and *T* has the units of temperature. Therefore, the entropy change has the units of energy divided by units of temperature. Sometimes, you might find the subscript “rev” in the Clausius definition which means that equation 1.1 is valid only for a “reversible” process. This is unnecessary. In fact, the additional requirement that the process be “reversible” may be even confusing since the term “reversible” (or irreversible) has many quite different meanings.

The quantity of heat, dQ must be very small, such that when it is transferred into, or out from the system, the temperature *T* does not change. If dQ is a finite quantity of heat, and one transfers it to a system which is initially at a given *T*, the temperature of the system might change, and therefore the change in entropy will depend on both the initial and the final temperature of the system. Note, that this equation does not define entropy but only changes in entropy for a particular process, i.e., a small exchange of heat (dQ>0 means heat flows into the system, dQ<0 means heat flows out of the system. Correspondingly, *dS* will be positive or negative when heat flows into or out from the system, respectively). There are many processes which do not involve heat transfer, yet, from Clausius’s definition and the postulate that the entropy is a state function, one could devise a path, leading from one state to another for which the entropy change can be calculated. For some numerical examples [24,26].

It should be remembered that during the time of Clausius the meaning of entropy was not clear. It was a well-defined quantity, and one could calculate changes in entropy for many processes without bothering with the meaning of entropy. In fact, there are many scientists who use the concept of entropy successfully and who do not care for its meaning, or whether if it has a meaning at all.

Entropy is useful quantity in chemical thermodynamics and engineering regardless of what it means on a molecular level.

To summarize, Clausius’s definition in Equation (1) requires the system to be at thermal equilibrium. However, in order to calculate finite changes in entropy one must carry out the process through a very large number of small steps, while the system is maintained at equilibrium.

### 1.2. Boltzmann’s Definition Based on the Total Number of Micro-States

Towards the end of the 19th century the majority of scientists believed that matter consists of small units called atoms and molecules. A few persistently rejected that idea arguing that there was no proof of the existence of atoms and molecules; no one has ever seen an atom.

On the other hand, the so-called kinetic theory of heat which was based on the assumption of the existence of atoms and molecules had scored a few impressive gains. First, the pressure of a gas was successfully explained as arising from the molecules bombarding the walls of the container. Then came the interpretation of temperature in terms of the kinetic energy of the molecules which was a remarkable achievement that supported and lent additional evidence for the atomic constituency of matter. While, the kinetic theory of heat was successful in explaining the concepts of pressure, temperature and heat, entropy was left lagging behind.

Boltzmann defined the entropy in terms of the total of number accessible micro-states of a system consisting of a huge number of particles, but characterized by the macroscopic parameters of energy *E*, volume *V* and number of particles *N*.

What are these “number of micro-states,” and how are they related to entropy?

Consider a gas consisting of *N* simple particles in a volume *V*, each particle’s micro-state may be described by its location vector $R_{i}$ and its velocity vector $v_{i}$. By simple particles we mean particles having no internal degrees of freedom. Atoms, such as argon, neon and the like are considered as simple. They all have internal degrees of freedom but these are assumed to be unchanged in all the processes we discuss here. Assuming that the gas is very dilute so that interactions between the particles can be neglected, then, all the energy of the system is simply the sum of the kinetic energies of all the particles.

Imagine that you have microscopic eyes, and you could see the particles rushing incessantly, colliding with each other, and with the walls from time-to-time. Clearly, there are infinite configurations or arrangements, or micro-states of the particles which are consistent with the requirements that the total energy is a constant, and that they are all contained within the box of volume *V*. Each particle is specified by its location $R_{i}$ and its velocity $v_{i}$. Thus, in classical mechanics, a description of all the locations and all the velocities of the particles consist of a micro-state of the system. In quantum mechanics one usually defines *W*, as the total number of quantum mechanical solutions of the Schrödinger equation for a system described by its energy *E,* its volume *V* and the total number of particle *N.* We use the shorthand notation (E,V,N) to describe the macro-state of the system.

Without getting bogged down with the question of how to estimate the total number of arrangements, it is clear that this is a huge number, far “huger” than the number of particles which is of the order $N\approx 10^{23}$. Boltzmann postulated the relationship which is now known as the Boltzmann entropy,

(2) $$S=k_{B}\log W$$

where $k_{B}$ is a constant, now known as the Boltzmann constant (1.380 × 10^−23^ J/K), and *W* is the number of accessible micro-states of the system. Here, log is the natural logarithm. At first glance, Boltzmann’s entropy seems to be completely different from Clausius’s entropy. Nevertheless, in all cases for which one can calculate changes of entropy one obtains agreements between the values calculated by the two methods.

Boltzmann’s entropy was not easy to swallow, not only by those who did not accept the atomic theory of matter, but also by those who accepted it. The criticism was not focused so much on the definition of entropy, but rather on the formulation of the Second Law of Thermodynamics. Boltzmann explained the Second Law as a probabilistic law. In Boltzmann’s words [28,29,30]:

“*… a system…when left to itself, it rapidly proceeds to disordered, most probable state.*”

“*Most probable state*.” This statement was initially shocking to many physicists. Probability was totally foreign to physical reasoning. Physics was built on the foundation of deterministic and absolute laws, no provisions for exceptions. The macroscopic formulation of the Second Law was absolute—no one has ever observed a single violation of the Second Law. Boltzmann, on the other hand, insisted that the Second Law is only statistical, entropy increases *most* of the time, not *all* the time. The decrease in entropy is not an impossibility but is only highly improbable.

There is another quantity which is sometimes also referred to as Boltzmann’s entropy. This is the H-function. We shall discuss this quantity in the next section.

Boltzmann’s entropy, as defined in Equation (2), has raised considerable confusion regarding the question of whether entropy is, or isn’t a subjective quantity.

One example of this confusion which features in many popular science books is the following: Entropy is assumed to be related to our “knowledge” of the state of the system. If we “know” that the system is at some specific state, then the entropy is zero. Thus, it seems that the entropy is dependent on whether one knows or does not know in which state (or states) the system is.

This confusion arose from misunderstanding *W* which is the total number of accessible micro-states of the system. If W=1, then the entropy of the system is indeed zero (as it is for many substances at 0 K). However, if there are *W* states and we know in which state the system is, the entropy is still k ln W and not zero! This kind of argument led some authors to reject the “informational interpretation” of entropy. For details and examples, see References [24,26].

Although, it was not explicitly stated in the definition, the Boltzmann entropy applies to an equilibrium state. In statistical mechanics this entropy applies to the so-called micro-canonical ensemble, i.e., to systems having a fixed energy *E*, volume *V*, and number of particles *N* (*N* could be a vector comprising the number of particles of each species $(N_{1},\ldots ,N_{c}$). It is also clear that the entropy of an ideal gas applies to a well-defined system at equilibrium.

In general, the Boltzmann entropy does not provide an explicit entropy function. However, for some specific systems one can derive an entropy function based on Boltzmann’s definition. The most famous case is the entropy of an ideal gas, for which one can derive an explicit entropy function. This function was derived by Sackur and by Tetrode in 1912 [31,32], by using the Boltzmann definition of entropy. This function was re-derived based on Shannon’s measure of information. We shall discuss this in Section 2.

### 1.3. ABN’s Definition of Entropy Based on Shannon’s Measure of Information

In this section we first present the so-called Shannon Measure of Information (SMI). Then we present an outline of the steps leading from SMI to Entropy. This is a relatively recent definition of entropy. It was originally used as an interpretation of entropy [22,24], but later turned into a definition of entropy. This definition is superior to both the Clausius and the Boltzmann definitions. Unlike the Clausius definition, which provides only a definition of changes in entropy, the present one provides the entropy function itself. Unlike Boltzmann’s definition, which is strictly valid for isolated systems and does not provide a simple intuitive interpretation, the present one is more general and provides a clear, simple and intuitive interpretation of entropy. It is more general in the sense that it relates the entropy to probability distributions, rather than to the number of micro-states. One final “bonus” is afforded by this definition of entropy. It does not only remove any trace of mystery associated with entropy, but it also expunges the so-called irreversibility paradox.

In this section we shall not discuss the Shannon measure of information (SMI) in any details. For details, see References [24,26]. We shall only quote the definition of SMI then outline the procedure to obtaining the entropy from the SMI.

We shall see that the entropy is, up to a multiplicative constant, nothing but a particular case of SMI. For more details, see References [22,24].

In 1948, Shannon published a landmark article titled, “A Mathematical Theory of Communication.” [33].

Here is how Shannon introduced the measure of information. In Section 6 of the article titled: “Choice, Uncertainty and Entropy,” we find:

*Suppose we have a set of possible events whose probabilities of occurrence are*$p_{1},p_{2},\cdots ,p_{n}$. *These probabilities are known but that is all we know concerning which event will occur. Can we find a measure of how much “choice” is involved in the selection of the event or how uncertain we are of the outcome?*

*If there is such a measure, say,*$H\left(p_{1},p_{2},\ldots ,p_{n}\right)$, it is reasonable to require of it the following properties:

1. *H should be continuous in the*$\;p_{i}$.
2. *If all the*$p_{i}$*are equal,*$p_{i}=\frac{1}{n}$, *then H should be a monotonic increasing function of n. With equally likely events there is more choice, or uncertainty, when there are more possible events.*
3. If a choice be broken down into two successive choices, the original H should be the weighted sum of the individual values of H.

Then Shannon proved the theorem:

The only *H* satisfying the three assumptions above has the form:

(3) $$H=-K\sum p_{i}\log p_{i}$$

Shannon did not seek a measure of the general concept of information, only a measure of information contained in, or associated with a probability distribution. This is an important point that one should remember whenever using the term “information” either, as a measurable quantity, or in connection with the Second Law of Thermodynamics.

We are ready to *define* the concept of entropy as a special case of SMI.

In this section we use for convenience the natural logarithm $\log_{e}x$, or lnx. Whenever we want to convert to SMI we need to multiply by $\log_{2}e$, i.e., $\log_{2}x=\log_{2}e{\;\log}_{e}x$. The overall plan of obtaining the entropy of an ideal gas from the SMI consists of four steps:

First, we calculate the *locational* SMI associated with the equilibrium distribution of locations of all the particles in the system.

Second, we calculate the velocity SMI associated with the equilibrium distribution of velocities (or momenta) of all the particles.

Third, we add a correction term due to the quantum mechanical *uncertainty principle*.

Fourth, we add a correction term due to the fact that the particles are *indistinguishable*.

**First step: The locational SMI of a particle in a 1D box of length *L***

Suppose we have a particle confined to a one-dimensional (1D) “box” of length *L*. Since there are infinite points in which the particle can be within the interval (0, *L*), the corresponding locational SMI must be infinite. However, we can define, as Shannon did, the following quantity by analogy with the discrete case:

(4)               H(X)=−∫f(x)logf(x)dx

This quantity might either converge or diverge, but in any case, in practice we shall use only differences between such quantities. It is easy to calculate the density distribution which maximizes the locational SMI, H(X) in Equation (4). The result is:

(5) $$f_{eq}\left(x\right)=\frac{1}{L}$$

This is a uniform distribution. The use of the subscript *Equation* (for equilibrium) will be clarified later. The corresponding SMI calculated by substituting Equation (5) in Equation (4) is:

(6)                                H(locations in 1D)=logL

We now acknowledge that the location of the particle cannot be determined with absolute accuracy, i.e., there exists a small interval $h_{x}$ within which we do not care where the particle is. Therefore, we must correct Equation (6) by subtracting $\log h_{x}$. Thus, we write instead of Equation (6):

(7) $$H\left(X\right)=\log L-\log h_{x}$$

We recognize that in Equation (7) we effectively defined H(X) for the *finite* number of intervals n=L/h. Note that when $h_{x}\to 0,\;H\left(X\right)$ diverges to infinity. Here, we do not take the mathematical limit, but we stop at $h_{x}$ small enough but not zero. Note also that in writing Equation (7) we do not have to specify the units of length for as long as we use the same units for *L* and $h_{x}$.

**Second step: The velocity SMI of a particle in a 1D “box” of length *L***

The mathematical problem is to calculate the probability distribution that maximizes the continuous SMI which is subject to two conditions, a normalization condition and a constant variation. For details see reference [34].

The result is the Normal distribution:

(8) $$f_{eq}\left(x\right)=\frac{\exp\left[-x^{2}/2\sigma^{2}\right]}{\sqrt{2\pi \sigma^{2}}}$$

The subscript eq which stands for equilibrium will be clarified once we realize that this is the equilibrium distribution of velocities. Applying this result to a classical particle having average kinetic energy $\frac{m<v_{x}^{2}>}{2}$, and using the relationship between the standard deviation $\sigma^{2}$ and the temperature of the system:

(9) $$\sigma^{2}=\frac{k_{B}T}{m}$$

We get the equilibrium velocity distribution of one particle in a 1D system,

(10) $$\;\;f_{eq}\left(v_{x}\right)=\sqrt{\frac{m}{2\pi k_{B}T}}\;\exp\left[\frac{-mv_{x}^{2}}{2k_{B}T}\right]$$

where $k_{B}$ is the Boltzmann constant, *m* is the mass of the particle, and *T* the absolute temperature. The value of the continuous SMI for this probability density is,

(11) $$H_{max}\left(velocity\;in\;1D\right)=\frac{1}{2}\log\left(2\pi ek_{B}T/m\right)$$

Similarly, we can write the momentum distribution in 1D, by transforming from $v_{x}\to p_{x}=mv_{x}$, to get,

(12) $$\;\;\;\;\;\;\;\;\;f_{eq}\left(p_{x}\right)=\frac{1}{\sqrt{2\pi mk_{B}T}}\;\exp\left[\frac{-p_{x}^{2}}{2mk_{B}T}\right]$$

and the corresponding maximum SMI:

(13) $$H_{max}\left(momentum\;in\;1\;D\right)=\frac{1}{2}\log\left(2\pi emk_{B}T\right)$$

As we have noted in connection with the locational SMI, we again recognize the fact that there is a limit to the accuracy within which we can determine the velocity (or the momentum) of the particle. We, therefore, correct the expression in Equation (13) by subtracting log $h_{p}$ where $h_{p}$ is a small, but finite interval:

(14) $$H_{max}\left(momentum\;in\;1D\right)=\frac{1}{2}\log\left(2\pi emk_{B}T\right)-\log h_{p}$$

Note, again, that if we choose the units of $h_{p}$ of momentum as mass length/time, the same as of $\sqrt{mk_{B}T}$, then the whole expression under the logarithm will be a pure number.

**Third step: Combining the SMI for the location and momentum of one particle; introducing the uncertainty principle**

In the previous two subsections, we derived the expressions for the locational and the momentum SMI of one particle in 1D system. We now combine the two results. Assuming that the location and the momentum (or velocity) of the particles are independent events we write:

(15) $$\begin{array}{c} H_{max}\left(location\;and\;momentum\right)=H_{max}\left(location\right)+\\ H_{max}\left(momentum\right)=\log\left[\frac{L\sqrt{2\pi emk_{B}T}}{h_{x}h_{p}}\right] \end{array}$$

Recall that $h_{x}$ and $h_{p}$ were chosen to eliminate the divergence of the SMI. In writing Equation (15) we assume that the location and the momentum of the particle are independent. However, quantum mechanics impose restrictions on the accuracy in determining both the location *x* and the corresponding momentum $p_{x}$. We must acknowledge that nature imposes on us a limit on the accuracy with which we can determine simultaneously the location and the corresponding momentum. Thus, in equation Equation (15), $h_{x}$ and $h_{p}$ cannot both be arbitrarily small, but their product must be of the order of Planck constant $h=6.626\times 10^{-34}$ J s. Thus, we set:

$h_{x}h_{p}\approx h,$ and instead of Equation (15), we write:

(16) $$H_{max}\left(location\;and\;momentum\right)=\log\left[\frac{L\sqrt{2\pi emk_{B}T}}{h}\right]$$

The SMI of a particle in a box of volume *V*

We consider again one simple particle in a cubic box of volume *V*. We assume that the location of the particle along the three axes *x*, *y* and *z* are independent. Therefore, we can write the SMI of the location of the particle in a cube of edges *L*, and volume *V* as:

(17) $$H\left(location\;in\;3D\right)=3H_{max}\left(location\;in\;1D\right)=3\log\;L=\log\;V$$

Similarly, for the momentum of the particle we assume that the momentum (or the velocity) along the three axes *x*, *y* and *z* are independent. Hence, we write:

(18) $$H_{max}\left(momentum\;in\;3D\right)=3H_{max}\left(momentum\;in\;1D\right)$$

We combine the SMI of the locations and momenta of one particle in a box of volume *V*, taking into account the uncertainty principle. The result is:

(19) $$H_{max}\left(location\;and\;momentum\;in\;3D\right)=3\log\left[\frac{L\sqrt{2\pi emk_{B}T}}{h}\right]$$

**Step four: The SMI of locations and momenta of *N* independent and indistinguishable particles in a box of volume *V***

The next step is to proceed from one particle in a box to *N* independent particles in a box of volume *V*. Given the location (x,y,z), and the momentum $\left(p_{x},p_{y},p_{z}\right)$ of one particle within the box, we say that we know the micro-state of the particle. If there are *N* particles in the box, and if their micro-states are independent, we can write the SMI of *N* such particles simply as *N* times the SMI of one particle, i.e.,:

(20) SMI( N independent  particles)=N×SMI(one particle)

This equation would have been correct if the micro-states of all the particles were independent. In reality, there are always correlations between the micro-states of all the particles; one is due to the indistinguishability between the particles. The second is due to *intermolecular interactions* between the particles. We shall mention here only the first correction due the indistinguishability of the particles. For indistinguishable particles we must correct Equation (20) and write instead:

(21) H(N indistinguishable particles)=Nlog V(2πmekBTh2)32−logN!

Using the Stirling approximation for logN! (note again that we use here the natural logarithm) in the form:

(22) logN!≈Nlog N−N

We have the final result for the SMI of *N* indistinguishable particles in a box of volume *V*, and temperature *T*:

(23) H(1,2,…N)=Nlog [VN(2πmkBTh2)32]+52N

This is a remarkable result. By multiplying the SMI of *N* particles in a box of volume *V*, at temperature *T*, by a constant factor ($k_{B}$, if we use the natural log, or $k_{B}\log_{e}2$ if the log is to the base 2), one gets the entropy, the thermodynamic entropy of an ideal gas of simple particles. This equation was derived by Sackur and by Tetrode in 1912, by using the Boltzmann definition of entropy. Here, we have derived the entropy function of an ideal gas from the SMI.

One can convert this expression to the entropy function S(E,V,N), by using the relationship between the total kinetic energy of the system, and the total kinetic energy of all the particles:

(24) $$E=N\frac{mv^{2}}{2}=\frac{3}{2}Nk_{B}T$$

The explicit entropy function of an ideal gas is obtained from Equations (23) and (24):

(25) S(E,V,N)=NkBln[VN(EN)32]+32kBN[53+ln(4πm3h2)]

We can use this equation as a definition of the entropy of an ideal gas of simple particles characterized by constant energy, volume and number of particles. Note that when we combine all the terms under the logarithm sign, we must get a dimensionless quantity. It should be noted that this entropy function is monotonically increasing function of *E*, *V* and *N*, and the curvature is negative with respect to each of these variables. This is an important property of the entropy function, which we will not discuss here. For more details, see [22,24].

To summarize, in the procedure of obtaining the entropy we started with the SMI associated with the locations and momenta of the particles. We calculated the distribution of the locations and momenta that maximizes the SMI. We referred to this distribution as the equilibrium distribution. The reason is that we know that for ideal gases, the distribution that maximizes the SMI is the same as the distribution at equilibrium. This is actually the experimental distribution of locations and momenta at equilibrium. Let us denote this distribution of the locations and momenta of all the particles by $f_{eq}\left(R,p\right)$.

Next, we use the equilibrium distribution to calculate the SMI of a system of *N* particles in a volume *V*, and at temperature *T*. This SMI is, up to a multiplicative constant $\left(k_{B}\ln2\right),$ identical to the entropy of an ideal gas at equilibrium. This also justifies the reference to the distribution which maximizes the SMI as the equilibrium distribution.

It should be noted that in the derivation of the entropy we used the SMI twice; first, in calculating the distribution that maximizes the SMI, then in evaluating the maximum SMI corresponding to this distribution. The distinction between the concepts of SMI and entropy is absolutely essential. Referring to the SMI (as many do) as entropy inevitably leads to such an awkward statement. The maximum value of the entropy (meaning the SMI) is the entropy (meaning the thermodynamic entropy). The correct statement is that the SMI associated with locations and momenta is defined for any system; small or large, at equilibrium or far from equilibrium. This SMI, not the entropy, evolves into a maximum value when the system reaches equilibrium. At this state, the SMI becomes proportional to the entropy of the system. The entropy obtained in this procedure is referred to as the Shannon based, or the ABN definition of entropy.

Since the entropy is up to a constant a special case of an SMI, it follows that whatever interpretation one accepts for the SMI, it will be automatically applied to the concept of entropy. The most important conclusion of this definition is that entropy, being a state function, is not a function of time. Entropy does not change with time, and entropy does not have a tendency to increase. It is very common to say that entropy increases towards its maximum at equilibrium. This is wrong. The correct statement is: The entropy is the maximum! As such it is not a function of time.

It is now crystal clear that none of the definitions of entropy hints on any possible time dependence of the entropy. This aspect of entropy stands out most clearly from the third definition.

It is also clear that all the equivalent definitions render entropy a state function; meaning that entropy has a unique value for a well-defined thermodynamic system.

Finally, we add a comment on the concept of equilibrium. We understand that there exist states in which the thermodynamic parameters, say temperature, pressure, or density do not change with time. These states are called equilibrium states.

Unfortunately, there is no general definition of equilibrium which applies to all systems. Callen [35,36], introduced the existence of the equilibrium state as a postulate. He also emphasized that any definition of an equilibrium state is necessarily circular.

In practice, we find many systems in which the parameters describing the system seem to be unchanged with time. Yet, they are not equilibrium states. But for all our purposes in this book, we can assume that every well-defined system, say having a fixed energy *E*, volume *V*, and number of particles *N* will tend to an equilibrium state. At this state, the entropy of the system, as well as many other thermodynamic relationships are applicable.

## 2. How Did Entropy Became Associated with Time

Open any serious textbook on thermodynamics, and you will find that Entropy is a state function. This means that entropy is defined for equilibrium states. Therefore, it is clear that it has nothing to do with time. Yet, surprisingly many authors associate entropy with time, and some even equate entropy to time.

The association of entropy with the Arrow of Time has been discussed in great detail in my books: “The Briefest History of Time,” and “Time’s Arrow; The Timeless Nature of Entropy and the Second Law” [11,27]. In this Section, I will summarize my views on the origin of, and the reasons for this distorted association of entropy with time. I will also present some outstanding quotations from the literature. More details are provided in the reference [34].

Open any book written by physicists which deals with a “Theory of Time,” “Time’s beginning,” and “Time’s ending,” and you will most likely find a lengthy discussion about association of entropy and the Second Law of Thermodynamics with time. The origin of this association of “Time’s Arrow” with entropy can be traced to Clausius’ s famous statement of the Second Law:

“*The entropy of the universe always increases.*”

The statement “*entropy always increases,*” always means that “*entropy always increases with time*.” Statements like “*entropy always increases*” are meaningless; entropy, in itself does not have a numerical value, therefore one cannot claim that it increases or decreases. Entropy changes are meaningful only for well-defined processes that occur in well-defined thermodynamic systems for which the entropy is defined; once we specify the system, then its entropy is determined and does not change with time.

As we have pointed out in Section 1 there is no indication whatsoever, from any definition of entropy that it changes with time. There is one exception which is the Boltzmann’s H-function, which as we shall soon show is not entropy. Why did Clausius conclude that entropy of the universe always increases? It is difficult to answer. Perhaps, Clausius imagined that there exists some nebulous abstract quantity out there or everywhere in the universe which always increases. The simpler answer is that Clausius did not, and in fact could not understand what entropy is on a molecular level, and his statement of the Second Law may be said to be an unwarranted generalization.

The concept of Time’s Arrow and its explicit association with the Second Law is attributed to Eddington, see Section 2.1. However, the association of the Second Law with time can be traced back to Boltzmann who related the one-way property of time with the Second Law, see Section 2.2.

### 2.1. Eddington’s Contribution to the Mess

As we stated in Section 1, the origin of the association of entropy with time is due to Clausius. However, the explicit association of entropy with the Arrow of Time is due to Arthur Eddington [19].

There are two very well-known quotations from Eddington’s (1928) book, “The Nature of the Physical World.” One concerns the role of entropy and the Second Law, and the second, introduces the idea of “time’s arrow.”

*“The law that entropy always increases, holds, I think, the supreme position among the laws of Nature. If someone points out that your pet theory of the universe is in disagreement with Maxwell’s equations—then so much the worse for Maxwell’s equations. If it is found to be contradicted by observation—well, these experimentalists do bungle things sometimes. But if your theory is found to be against the second law of thermodynamics I can give you no hope; there is nothing for it but to collapse in deep humiliation.”*

*“Let us draw an arrow arbitrarily. If as we follow the arrow we find more and more of the random element in the state of the world, then the arrow is pointing towards the future; if the random element decreases the arrow points towards the past. That is the only distinction known to physics. This follows at once if our fundamental contention is admitted that the introduction of randomness is the only thing which cannot be undone. I shall use the phrase ‘time’s arrow’ to express this one-way property of time which has no analogue in space.”*

In the first quotation, Eddington reiterates the unfounded idea that “entropy always increases.” Although I agree that the Second Law of thermodynamics is unique compared with other laws of physics, I do not agree with the statement that “entropy always increases.”

Although, it is not explicitly stated, the second alludes to the connection between the Second Law and the Arrow of Time. This is clear from the association of the “random element in the state of the world” with the “arrow pointing towards the future.”

In my view it is far from clear that an Arrow of Time exists [34]. It is also clear that entropy is not associated with randomness, and it is far from clear that entropy always increases.

Therefore, my conclusion is that entropy has nothing to do with time!

Of course, I am well aware of many statements in the literatures which identify entropy with time. I have written about this topic in my books [11,27,34]. I will bring here a few more quotations from popular science books:

In Rifkin’s book (1980) [16] we find:

*“…the second law. It is the irreversible process of dissipation of energy in the world. What does it mean to say, ‘The world is running out of time’? Simply this: we experience the passage of time by the succession of one event after another. And every time an event occurs anywhere in this world energy is expended and the overall entropy is increased. To say the world is running out of time then, to say the world is running out of usable energy. In the words of Sir Arthur Eddington, ‘Entropy is time’s arrow.”*

It is NOT!

There are many other statements in Eddington’s book which are unfounded and misleading. For instance; the claim that entropy is a subjective quantity, the concepts of “*entropy-clock*,” and “*entropy-gradient*.” Reading through the entire book by Eddington, I did not find a single correct statement on the thermodynamic entropy! More on that in Reference [27].

It is strange to me that most people who write about entropy, specifically in popular science books quote Eddington’s book as the origin of the “great idea” connecting entropy and the Second law with Time’s Arrow.

Another shameful example of associating entropy with time is Brillouin’s (1962) [37]:

*“The probability has a natural tendency to increase, and so does entropy.”*

This single sentence contains three wrong ideas! Probability (of what?) has no “natural tendency to increase”! Entropy (of what?) has no “natural tendency to increase”! and Probability does not behave as entropy!

Claiming that entropy has a tendency to increase is wrong and meaningless. Saying that probability has a tendency to increase is tantamount to saying that beauty, wisdom or stupidity has a tendency to increase. Combining entropy and probability without referring to any specific system or event is shameful! Nothing less than that.

Similarly, the common statement of the Second Law: “Entropy always increases,” is meaningless unless one specifies the system for which the entropy is discussed. Once the system is specified, its entropy is fixed and does not change with time.

Such statements were driven to the extremes of absurdity by using the quality sign “=” in order to express the identity of time’s arrow and entropy. In “The Demon and the Quantum,” Scully writes [13]:

*“The statistical time concept that entropy = time’s arrow has deep and fascinating implications. It therefore behooves us to try to understand entropy ever more deeply. Entropy not only explains the arrow of time; it also explains its existence; it is time.”*

### 2.2. Boltzmann’s H-Theorem; Its Criticisms, Boltzmann’s Answers and the Seeds of an Enormous Misconception About Entropy

In 1877 Boltzmann proved a truly remarkable theorem known as the H-theorem. He defined a function H(t), and proved that it decreases with time and reaches a minimum at equilibrium.

In the following I shall briefly present the H-theorem, the main criticism, and Boltzmann’s answer. We will point out where Boltzmann went wrong and why the function −H(t) is not entropy, and why the H-theorem does not represent the Second Law. More details in reference [34].

**Boltzmann’s H-theorem**

Boltzmann defined a function H(t) as:

(26) H(t)=∫f(v,t)log[f(v,t)]dv

In proving the H-theorem, he made the following assumptions:

1. Ignoring the molecular structure of the walls (perfect smooth walls).
2. Spatial homogenous system or uniform locational distribution.
3. Assuming binary collisions, conserving momentum and kinetic energy.
4. No correlations between location and velocity (assumption of molecular chaos).

Details of the assumptions and the proof of the theorem can be found in many textbooks. Basically, Boltzmann proved that,

(27) $$\frac{dH\left(t\right)}{dt}\leq 0$$

and at equilibrium, i.e., t→∞,

(28) $$\frac{dH\left(t\right)}{dt}=0\;\;$$

Boltzmann believed that the behavior of the function −H(t) is the same as that of the entropy, i.e., the entropy always increases with time, and at equilibrium reaches a maximum. From then on, the entropy does not change with time.

This theorem drew great deal of criticism, the most well-known of which are:

*I*. The “Reversal Paradox”

The *H*-theorem singles out a preferred direction of time. This is inconsistent with the time reversal invariance of the equations of motion.

In other words, one cannot obtain time-asymmetric behavior from time-symmetric equations of motion.

*II*. *The “Recurrence Paradox”, Based on Poincare’s Theorem*

After a sufficiently long time an isolated system with fixed *E, V*, *N*, will return to an arbitrary small neighborhood of almost any given initial state.

If we assume that dH/dT<0 at all *t,* then obviously *H* cannot be a periodic function of time.

Both paradoxes seem to arise from the conflict between the reversibility of the equations of motion on one hand, and the apparent irreversibility of the Second Law, namely that the H-function decreases monotonically with time, on the other hand. Boltzmann rejected the criticism by claiming that H does not always decrease with time but only with high probability. The irreversibility of the Second Law is not absolute, but only highly improbable. The answer to the recurrence paradox follows from the same argument. Indeed, the system can return to the initial state. However, the recurrence time is so large that this is never observed, not in our lifetime, not even in the life time of the universe.

Notwithstanding Boltzmann’s correct answers to his critics, Boltzmann and his critics made an enduring mistake in the interpretation of the H-function, a lingering mistake that has hounded us ever since. This is the very identification of the function −H(t) with the behavior of the entropy. This error has been propagated in literatures to this day.

Boltzmann was right in claiming that the reversal of the state of the system is not impossible but highly improbable. Boltzmann was also right in claiming that the H(t) function can go up and can fluctuate even after reaching an equilibrium state. In fact, the H function can even reach the initial value.

Unfortunately, Boltzmann was wrong in believing that the entropy can go down; “not impossible, but highly improbable.” The entropy of the system like the summit of the hill, cannot go down, nor reach the initial state. In order for the entropy to decrease to its value at the initial state, the system must visit the initial state, and stay there at equilibrium.

It is clear from the very definition of the function H(t), that −H(t) is a SMI, and if one identifies the SMI with entropy then we go back to Boltzmann’s identification of the function −H(t) with entropy.

Fortunately, thanks to the recent derivation of the entropy function, i.e., the function S(E,V,N) based on the SMI, it becomes crystal clear that the SMI is not entropy! Specifically the function −H(t) is SMI based on the velocity distribution. Clearly, one cannot identify −H(t) with entropy. To obtain the entropy one must first define the −H(t) function based on the distribution of both the locations and momentum:

(29) −H(t)=−∫f(R,p,t)logf(R,p,t)dRdp

This function may be defined for a system at equilibrium, or very far from equilibrium. To obtain the entropy we must take the maximum of −H(t) over all possible distributions f(R,p,t):

(30) $$\;\;\;Entropy=\underset{over\;all\;fs}{\max}\left[-H\left(t\right)\right]$$

We also believe that once the system attains an equilibrium, the −H(t) attains its maximum value, i.e., we identify the maximum over all possible distributions with the maximum of SMI in the limit t→∞:

(31) $$\;\;\;\;\;\;\;\;Entropy=\underset{t\to \infty }{\lim}\left[-H\left(t\right)\right]=Max\;SMI\;\left(at\;equilibrium\right).$$

At this limit we obtain the entropy (up to a multiplicative constant) which is clearly not a function of time!

Boltzmann, as well as his critics and many others believed that −H(t) is entropy, and the time dependence of −H(t) is the same as the (supposed) behavior of entropy, namely; that entropy always increases with time. This belief rests on the form of the H-function which resembles the form of the Gibbs entropy, as well as what is commonly referred to as Shannon’s entropy.

Thus, once it is understood that the function −H(t) is an SMI and not entropy, it becomes clear that the criticism of Boltzmann’s H-Theorem is addressed to the evolution of the SMI, and not to the entropy. At the same time, Boltzmann was right in defending his H-theorem when viewed as a theorem on the evolution of SMI, but he was wrong in his interpretation of the quantity −H(t) as entropy.

Boltzmann, as well as many others believed that the H-theorem represents the behavior of entropy, namely that *S* increases monotonically with time until reaching a maximum value.

Critics of Boltzmann’s theorem also believed that the entropy changes monotonically with time, and found a conflict between the H-theorem, on one hand, and the “time-reversal” of equations of motion, on the other hand. Boltzmann correctly answered his critics by claiming that −H(t) increases monotonically only with high probability, and that at equilibrium the entropy might decrease; it is not impossible, but highly improbable event.

Boltzmann’s answer was correct, for as long as it applies to −H(t) as a SMI. Boltzmann and his critics were wrong in interpreting −H(t) as entropy. Entropy *does not* change with time. It does not decrease spontaneously, not even with small probability.

This conclusion followed from the derivation of entropy from the SMI, and it is the essence of the “Timeless Nature of Thermodynamics.”

It should be noted that in some popular science books one finds figures showing how the entropy changes with time [5.12] but applied to the “entire universe.” This is of course, meaningless since the entropy of the universe is not definable.

The latter conclusion also dismisses Clausius’s formulation of the Second Law. Thus, the entropy of a well-defined thermodynamic system is timeless. The entropy of the universe is not timeless; it is simply meaningless.

It is quite common to present the H-theorem as a “proof of the “behavior of the entropy,” i.e., the increase of entropy with time, which for over a hundred years was considered to be the ultimate formulation of the Second Law. For instance, Davies (1995) writes:

*“He [Boltzmann] discovered a quantity, defined in terms of the motions of the molecules that provided a measure of the degree of chaos in the gas. This quantity, Boltzmann proved, always increases in magnitude as a result of the molecular collision, suggesting it be identified with thermodynamic entropy. If so, Boltzmann’s calculation amounted to a derivation of the second law of thermodynamics from Newton’s laws.”*

This is clearly not true. First, the quantity defined by Boltzmann is not a “measure of the degree of chaos.” Second, Boltzmann did not prove the Second Law from Newton’s laws. This is a typical erroneous statement of Boltzmann’s H-theorem which dominates the literatures.

### 2.3. The Most Ridiculous Idea, Called the “Past Hypothesis”

Most writers on entropy and the Second Law who adhere to Clausius’s statement of the Second Law claim that since the entropy of the universe always increases, it follows that the entropy of the universe must have been low at, or near, the Big Bang. This is known as the *Past Hypothesis*; a meaningless hypothesis suggested by Albert [1,5,27]. This hypothesis is both trivial and absurd.

It is trivial, since if you are told that “entropy always increases,” then it is trivial to conclude that the entropy yesterday was lower than today, and it was much lower at the Big Bang. It is absurd for the simple reason that there is no such a law that says that “entropy always increases.” Entropy, by itself has no numerical value. It can either increase or decrease for some specific processes in some specific systems. Thus, it easy to see why the Past Hypothesis is trivial. It is however, far from trivial to understand why it is absurd.

Carroll [5] even went a step further and used the Past Hypothesis to “*explain”* almost everything that happens including our ability to remember the past but not the future. Carroll dedicates a whole book endlessly repeating these meaningless ideas about the “entropy of the early universe” and its relationship with the Arrow of time. I have criticized Carroll’s book in more detail in [11,27]. Here, I will present only one quotation which is the most absurd of all the absurdities which I ever read, it is found in page 43 of Carroll’ book:

*When it comes to the past, however, we have at our disposal both our knowledge of the current macroscopic state of the universe, plus the fact that the early universe began in a low-entropy state. That one extra bit of information, known simply as the “Past Hypothesis,” gives us enormous leverage when it comes to reconstructing the past from the present.*

This ridiculous idea is endlessly repeated throughout Carroll’s book. What does it mean? Nothing! Why? Because of the following:

1. We do not know the macro-state of the Universe at present.
2. The entropy of the universe is not defined.
3. It is meaningless to talk about the entropy of the universe today, yesterday or a million years ago.
4. It is not known if the universe “began” at some time.
5. To assign entropy to the universe at some hypothetical time is meaningless
6. “That extra bit” is no bit at all.
7. “Enormous leverage” is already beyond description; it is, in fact a zero leverage.

Carroll not only parroted Albert’s faulty idea, but he took a meaningless idea, and inflated, and exaggerated it to enormous proportions! Do we know the fact (fact?) that the early universe began in a low-entropy state? This is not a fact, but a meaningless, fictitious nonsense!

Another unwarranted “prediction” may be referred to as the “Future Hypothesis” which basically states that the universe is doomed to “thermal death.” This hypothesis is also unwarranted. More on this in references [11,27,34].

### 2.4. Some Recent Amusing Ideas about Entropy and Time

Another amusing idea about the association of entropy with time, may be found in Müller’s recent book [38]:

*“Eddington attributed the flow of time to the increase in entropy, a measure of disorder in the universe. We now know enormously more about the entropy of the universe than did Eddington in 1928 when he proposed the theory, and I’ll argue that Eddington got it backward. The flow of time causes entropy to increase, not the other way around.*

There are several points with which I do not agree with. First, entropy is not a measure of “disorder” in the universe [11,34]. It is quite strange that this erroneous interpretation of entropy still appears in a book published in 2016.

Second, no one knows how to define the entropy of the universe. Therefore, the statement about our knowledge of the entropy of the universe either now, or in 1928 is highly misleading. We now know about the entropy of the universe, as much as we know about the wisdom or the stupidity of the universe.

Third, the statement that the flow of time causes entropy to increase and not the other way around is doubly misleading. Entropy, by itself, cannot be said to increase or decrease. The reason is that entropy is a state function, i.e., it is defined for a well-defined system at equilibrium. As such, it is not a function of time. The flow of time is not the cause of entropy increase! The “flow of time” (if it flows at all) has nothing to do with entropy increase!

In Rovelli’s book [39], *Seven Brief Lessons on Physics***,** we find on page 52:

*“Why does heat go from hot things to cold things, and not vice versa?”*

His answer:

*“It is a crucial question because it is related to the nature of time.”*

No, it has nothing to do with the “nature of time.”

It is strange that the author himself explains that the process of heat-transfer from a hot to a cold body is *statistical*, i.e., with high probability. As such, it only looks to us as being “always” in one direction, but in fact, it can also be in the other direction, i.e., heat can flow from a cold to a hot body. This event can occur with a very small probability.

There is another misleading statement on the same page:

*“The difference between the past and the future exists only when there is heat.”*

This is doubly misleading. First, because it says “where there is heat,” and that has nothing to do with time, and second, as he explains “there is heat,” means there is *heat flow* from a hot to a cold body. This seems to be only in one direction, but this is only an apparent one direction, and has nothing to do with time.

## 3. Conclusions

As we saw in Section 1, entropy is defined only for the equilibrium state. Entropy is a state function, and as such, it is not a function of time, and whenever it is defined, it does not change with time.

Boltzmann, as many others failed to pinpoint the *subject* on which probability operated. In other words, the failure to distinguish between the two statements:

1. When we remove a constraint, the *state* of the system changes with high probability to a new equilibrium state.
2. When we remove a constraint, the *entropy* of the system changes with high probability towards a maximum.

While, the first statement is correct, and in fact applies to any thermodynamic system (isolated, isothermal, isothermal isobaric, etc.), the second is incorrect. First, the entropy formulation of the Second Law applies only to isolated systems, and second, the correct formulation is that the SMI, defined on the distribution of locations and momenta of all particles in an isolated system tends to a maximum at equilibrium, and at equilibrium the value of the Maximum SMI is proportional to the entropy of the system.

Since there is only a single distribution that maximizes the SMI, it follows that the entropy of a thermodynamic system has a unique value. It does not change with time, it does not reach a maximum value with time, and it does not fluctuate at equilibrium (neither with low or high probability).

In many popular science books, one can find plots showing how entropy changes with time. Most of the time fluctuations are small, but once in many billions of years it might have a big fluctuation. This behavior should be ascribed to the SMI, and not to the entropy.

In conclusion one can say that the main reasons for the misconstrued association of entropy with time are the following:

1. Confusing Shannon’s measure of information with the thermodynamic entropy.
2. Confusing various concepts of reversibility and irreversibility.
3. Confusing reversal of the system to its initial state, with reversal of the value of the entropy of the system.

Thanks to the new definition of entropy based on Shannon’s Measure of Information, all the confusion was clarified and thus evaporated. The new definition of entropy shows unequivocally that entropy is not a function of time and does not have a “tendency to increase with time.”
